# A Dual Pathogenic Mechanism Links Tau Acetylation to Sporadic Tauopathy

**DOI:** 10.1038/srep44102

**Published:** 2017-03-13

**Authors:** Hanna Trzeciakiewicz, Jui-Heng Tseng, Connor M. Wander, Victoria Madden, Ashutosh Tripathy, Chao-Xing Yuan, Todd J. Cohen

**Affiliations:** 1 Department of Biochemistry and Biophysics, University of North Carolina at Chapel Hill, Chapel Hill, NC 27599, USA; 2 Department of Neurology, University of North Carolina, Chapel Hill, North Carolina 27599, USA; 3 Department of Pathology and Laboratory Medicine, University of North Carolina, Chapel Hill, North Carolina 27599, USA; 4 Alexion Pharmaceuticals Inc, New Haven, Connecticut 06510, USA; 5 Department of Neurology, UNC Neuroscience Center, University of North Carolina, Chapel Hill, North Carolina 27599, USA

## Abstract

Tau acetylation has recently emerged as a dominant post-translational modification (PTM) in Alzheimer’s disease (AD) and related tauopathies. Mass spectrometry studies indicate that tau acetylation sites cluster within the microtubule (MT)-binding region (MTBR), suggesting acetylation could regulate both normal and pathological tau functions. Here, we combined biochemical and cell-based approaches to uncover a dual pathogenic mechanism mediated by tau acetylation. We show that acetylation specifically at residues K280/K281 impairs tau-mediated MT stabilization, and enhances the formation of fibrillar tau aggregates, highlighting both loss and gain of tau function. Full-length acetylation-mimic tau showed increased propensity to undergo seed-dependent aggregation, revealing a potential role for tau acetylation in the propagation of tau pathology. We also demonstrate that methylene blue, a reported tau aggregation inhibitor, modulates tau acetylation, a novel mechanism of action for this class of compounds. Our study identifies a potential “two-hit” mechanism in which tau acetylation disengages tau from MTs and also promotes tau aggregation. Thus, therapeutic approaches to limit tau K280/K281 acetylation could simultaneously restore MT stability and ameliorate tau pathology in AD and related tauopathies.

Tau proteins form hallmark neurofibrillary tangles (NFTs) and other pathological lesions that characterize Alzheimer’s disease (AD) and related tauopathies. Six tau isoforms are present in the brain containing either three (3R-tau) or four (4R-tau) repeat domains that together with the flanking regions mediate physiological tau-microtubule (MT) binding and confer MT stability^1^,^2^. Tau phosphorylation in regions mostly flanking the MT-binding region (MTBR) is the dominant mechanism thought to control tau function. However, tau is subject to a myriad of other post-translational modifications (PTMs) (e.g. ubiquitination, acetylation, methylation, glycosylation, polyamination)^3^ that alter tau’s biochemical properties, indicating that a complex tau PTM profile coordinately regulates tau function, which could ultimately determine aberrant tau pathology in diseased brains.

We and others demonstrated that tau is subject to acetylation within the lysine-rich MTBR^4^,^5^,^6^,^7^,^8^,^9^. While the full complement of enzymes controlling tau acetylation is not well-characterized, *in vitro* and cell-based experiments showed that CREB-binding protein (CBP) or the highly homologous p300 acetylates tau with high affinity in the MTBR^4^,^8^,^10^. In the complete absence of CBP or p300, tau also possesses an intrinsic ability to self-acetylate similar to that observed with auto-regulated acetyltransferases (e.g. MYST-family), implying that the energetically favorable acetyl group transfer may occur if acetyl-CoA levels are present at sufficient concentrations in the cytoplasm where tau is enriched^10^. Counter-acting tau acetylation, the deacetylases HDAC6 and SIRT1 have been shown to deacetylate tau *in vitro* and in cultured cells, highlighting a potential neuroprotective role for tau deacetylation by HDACs/Sirtuins^4^,^8^.

Several studies have implicated tau acetylation in the formation and maturation of NFTs^4^,^6^,^8^,^11^,^12^. Using an acetylation-specific tau antibody recognizing residue K280, tau lesions were readily detected in all 4R-tauopathies analyzed including AD, corticobasal degeneration (CBD), progressive supranuclear palsy (PSP), and a panel of familial AD cases, but not 3R-tau predominant Pick’s disease (PD) lacking exon 10 that harbors the K280 residue^4^. Supporting a pathological role for tau acetylation, CBP-acetylated tau proteins composed solely of the MTBR (tau-K18) showed increased tau filament formation as assessed by sedimentation assays, elevated Thioflavin T (ThT) reactivity, and the formation of tau fibrils by electron microscopy (EM).

The affinity for particular acetylated lysine residues or the exact stoichiometry of tau acetylation may be critical determinants of tau aggregation^5^. However, the consistent detection of K280-acetylated insoluble tau in several tau transgenic mouse models^4^,^13^,^14^ and human tauopathy brain samples^4^,^6^,^11^,^12^ supports the notion that tau K280 acetylation increasingly correlates with tau pathogenesis. More recently, tau acetylation-mimicking mutations at other prominent sites including K174, K274, K281 induced a series of AD-like deficits including synaptic dysfunction, neuronal loss, and cognitive impairments in viral-transduced and transgenic mouse models, supporting an acetylation-mediated pathological cascade^15^,^16^. A detailed understanding of how acetylation regulates tau function could foster new avenues to limit acetylated tau as plausible therapeutic strategies.

Early biochemical studies showed that the positively charged tau residues K280 and K281 physically engage MTs, highlighting the double lysine containing PHF6* motif (^275^VQIINK/K^281^) in exon 10 as a hotspot for tau-MT binding in addition to promoting tau aggregation^17^. The importance of residue K280 is reflected by the genetic deletion of this residue in FTDP-17 class dementia (ΔK280)^18^ or by the acetylation of residues K280 or K281, as we observed in AD and other tauopathies^4^. Either lysine deletion or acetylation is predicted to neutralize the overall positive lysine charge within this region, potentially abrogating tau-MT binding. However, in contrast to the relatively rare genetic ΔK280 mutation, positive K280-acetylated lesions are observed in most sporadic 4R-tauopathies analyzed to date^11^,^12^ and may represent a common feature of AD and other tauopathies.

Here, we provide evidence that tau K280/K281 acetylation induces pathogenic hallmarks consistent with both loss and gain of tau-mediated toxicity. Surprisingly, we show that tau acetylation alters phosphorylation at residues S202/T205 (comprising the AT8 epitope), indicating acetylation-dephosphorylation cross-talk. Using a series of biochemical approaches, we found that K280/K281 acetylation impaired tau-mediated MT assembly function and also significantly enhanced tau aggregation. Our results highlight the PHF6* double lysine motif as a critical hotspot playing a dual role in normal and pathological tau function. Therefore, modulating the acetylation machinery to limit K280/K281 acetylation could provide therapeutic benefits in AD and other tauopathies.

## Results

### Tau acetylation modulates tau phosphorylation status

**Figure 1:**
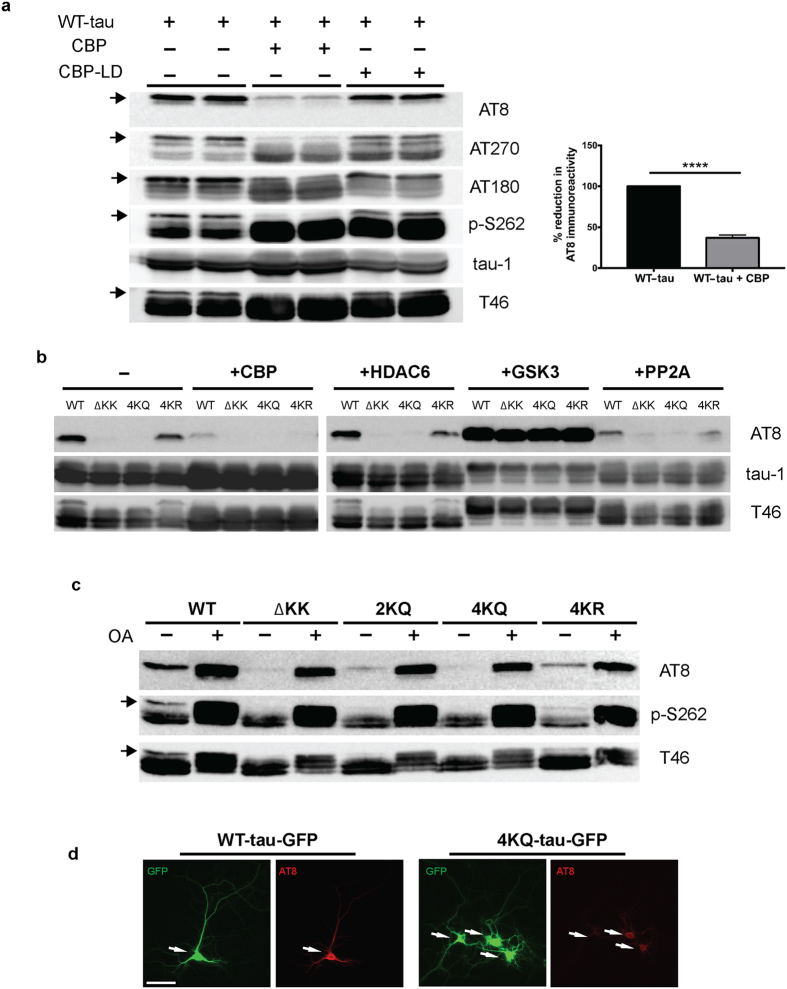
Tau acetylation modulates tau phosphorylation at specific epitopes. **(a–c**) Immunoblot analysis and quantification using the indicated tau antibodies for **(a**) cells expressing WT tau-T40 and either an active form of CBP or a catalytically inactive form of CBP (CBP-LD), (**b**) cells co- expressing tau-T40 (WT, ΔKK, 4KQ, or 4KR) and tau modifying enzymes, and (**c**) cells expressing tau-T40 (WT, ΔKK, 2KQ, 4KQ, or 4KR) and treated with control (DMSO) or okadaic acid (OA), where indicated. Solid black arrows highlight the ~75 kDa phospho-tau species that is reduced upon acetylation. Statistical significance was assessed using a student t-test (****p < 0.0001). Cropped images from full size immunoblots are provided in panels a–c. Full-length immunoblots are presented in Supplementary Fig. S1. (**d**) Immunofluorescence microscopy of primary cortical neurons expressing WT-tau-GFP (left) or 4KQ-tau-GFP (right) detecting phosphorylated tau (AT8). White arrows identify transfected neurons. Scale bar, 50 µm.

Full-length 2N4R-tau (also referred to as tau-T40) was co-expressed in cultured QBI-293 cells in the presence of wild-type CBP or a catalytically inactive CBP mutant (harboring L1435A/D1436A mutations, referred to as CBP-LD). Surprisingly, we observed that CBP-acetylated tau led to specific loss of the slower migrating ~75 kD tau species, initially detected using total tau antibodies, which potentially reflected reduced phosphorylation mediated by tau acetylation, an effect that was not observed with CBP-LD that is unable to facilitate tau acetylation (Fig. 1a, Supplementary Fig. S1). Subsequent immunoblotting showed near complete loss of AT8 immunoreactivity as well as loss of higher mobility ~75 kDa tau bands using a series of other phospho-tau antibodies.

Tau contains four prominent acetylation sites (K163, K280, K281, and K369) that were previously identified by mass spectrometry of immunopurified tau from cultured cells^4^. Thus, we introduced acetylation-mimics (4KQ), non-mimics (4KR), or a double lysine deletion (ΔK280/K281), which either maintain (e.g. K → R) or reduce (e.g. K → Q or ΔKK) the positive charge within this region. The tau proteins were expressed alone or in combination with a panel of enzymes that modify tau including acetyltransferases/deacetylases (CBP and HDAC6) and kinases/phosphatases (GSK3 and PP2A), and analyzed by immunoblotting (Fig. 1b and c, Supplementary Fig. S1). Similar to CBP-acetylated tau, acetylationmimic tau at either K280/K281 (2KQ) or all four lysines (4KQ), or surprisingly deletion of K280/K281 altogether (ΔKK), caused similar loss of S202/T205 phosphorylation (Fig. 1b and c). Supporting the involvement of PP2A phosphatases in acetylation-induced dephosphorylation, over-expression of a PP2A targeting subunit (PP2A-B56α) led to S202/T205 dephosphorylation (Fig. 1b), while the PP2A inhibitor okadaic acid (OA) restored S202/T205 immunoreactivity in cells expressing either ΔKK, 2KQ, or 4KQ mutants (Fig. 1c). Thus, either loss of the K280/K281 positive charge or their neutralization via acetylation is sufficient to promote dephosphorylation at the AT8 epitope.

To evaluate whether tau acetylation reduced AT8 immunoreactivity in neurons, we generated GFP-tagged versions of WT tau-T40 (WT-tau-GFP) or the 4KQ acetylation-mimic (4KQ-tau-GFP). As shown in Fig. 1d, WT tau expression in primary cortical neurons is pan-neuronal while AT8 immunoreactivity is predominantly restricted to the proximal axonal and somato-dendritic compartments, as previously noted^19^, likely reflecting enhanced PP2A activity in more distal regions of the axonal compartment. In contrast, expression of the acetylation-mimic 4KQ-tau showed reduced AT8-positive immunoreactivity, including within the somato-dendritic compartment of 4KQ-tau-GFP transfected neurons (Fig. 1d, see arrows). Thus, tau acetylation within the MTBR modulates distant tau phosphorylation status, potentially by generating a MT-dissociated cytosolic tau pool that is more susceptible to PP2A-mediated dephosphorylation. To evaluate whether tau acetylation leads to MT dissociation, we sought to examine downstream functional consequences on MT-assembly and pathological tau aggregation.

### Tau acetylation-mimics impair physiological MT assembly activity

**Figure 2:**
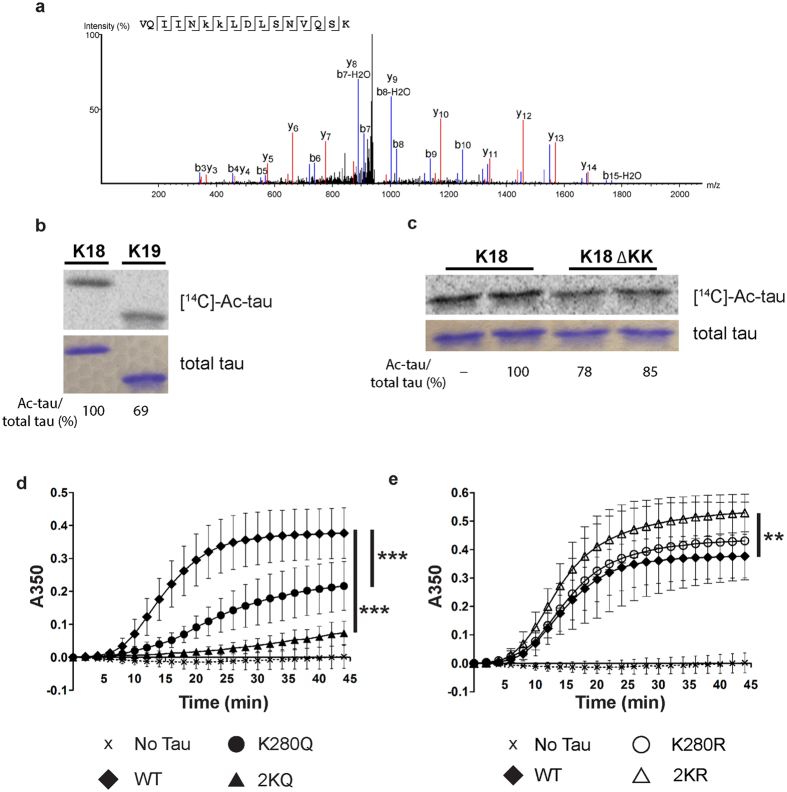
Tau acetylation-mimics impair tau-mediated MT assembly. **(a**) Peptide spectra depicting doubly acetylated K280 and K281 (^275^VQIIN**KK**LDLSNVQSK^290^) from CBP-acetylated tau proteins^4^. Ion scores are provided in Supplementary Fig. S2. (**b**,**c**) Autoradiography and Coomassie blue staining of (**b**) WT 4R-tau-K18 and 3 R-tau-K19 or **(c)** WT tau-K18 and tau-K18 ΔKK (containing deletion of residues K280 and K281). Cropped images from full size gels are provided in panels (**b**,**c**). Full-length gels are presented in Supplementary Fig. S3. (**d**,**e**) Light scattering assay in the presence of tau-K18 proteins detecting tubulin polymerization, as determined by absorbance readings at 350 nm (No Tau, X (N = 7); WT, ♦ (N = 9); K280Q, ● (N = 8); 2KQ, ▲ (N = 4); K280R, ◯ (N = 7); 2KR, Δ (N = 6)). Error bars indicate s.d. of the mean. Statistical significance was assessed using a student t-test as follows: WT vs. K280Q (***p < 0.0001); WT vs. K280R (ns, p = 0.36); K280Q vs. K280R (***p < 0.0001); WT vs. 2KR (**p < 0.0072); WT vs. 2KQ (***p < 0.0001); 2KQ vs. 2KR (***p < 0.0001).

Tau residues K280 and K281 within the PHF6* double lysine motif were shown to directly contact and stabilize charge interactions between tau and MTs^17^. Therefore, we hypothesized that K280/K281 acetylation, and hence neutralization of this positive charge, would impair MT stabilizing activity, leading to cytosolic accumulation and loss of normal tau function. First, we assessed whether both lysines K280 and K281 could be subject to acetylation by mass spectrometry analysis of CBP-acetylated full-length tau-T40 from cultured cells^4^. Indeed, in addition to singly acetylated peptides, we identified a prominent tryptic tau peptide in which K280/K281 were doubly acetylated, suggesting that K280/K281 are subject to either individual or tandem acetylation (Fig. 2a and Supplementary Fig. S2). *In vitro* acetylation reactions using radiolabeled [^14^C]-acetyl-CoA showed that the 3R-tau repeat domain (tau-K19), which lacks the 2^nd^ MTBR repeat containing residues K280/K281, or the 4R-tau repeat domain (tau-K18) containing a deletion of K280/K281 (referred to as ΔKK) led to slightly reduced overall tau acetylation levels (Fig. 2b and c, Supplementary Fig. S3), highlighting the K280/K281 residues as two of the more prominent acceptor sites among the >20 tau lysines that are subject to acetylation *in vitro*.

To further assess tau-MT regulation, we examined whether acetylation-mimic or non-mimic mutations at K280/K281, either singly or in combination, could alter MT assembly function. Tubulin polymerization reactions were performed using recombinant WT tau-K18 or K280/K281 mutants containing acetylation-mimic (K → Q) or non-mimic (K → R) mutations and MT assembly was assessed by optical density (O.D) readings (Fig. 2d)^20^. As expected, WT tau promoted tubulin assembly into polymerized MTs. In contrast, the single K280Q mutation impaired tau-mediated MT assembly by ~50%, while the double K280/281Q mutant showed near complete abrogation of MT assembly activity to baseline levels, similar to assembly reactions lacking tau altogether (Fig. 2d). Notably, the non-mimics containing single K280R or double 2KR mutations, which maintain the positive charge at residues K280/K281, showed either no effect or a mild enhancement of MT assembly kinetics (Fig. 2e). These results indicate that acetylation-mimics induce charge neutralization at K280/K281 and impair tau-mediated stabilization of MTs.

### Tau acetylation-mimics promote tau aggregation

**Figure 3:**
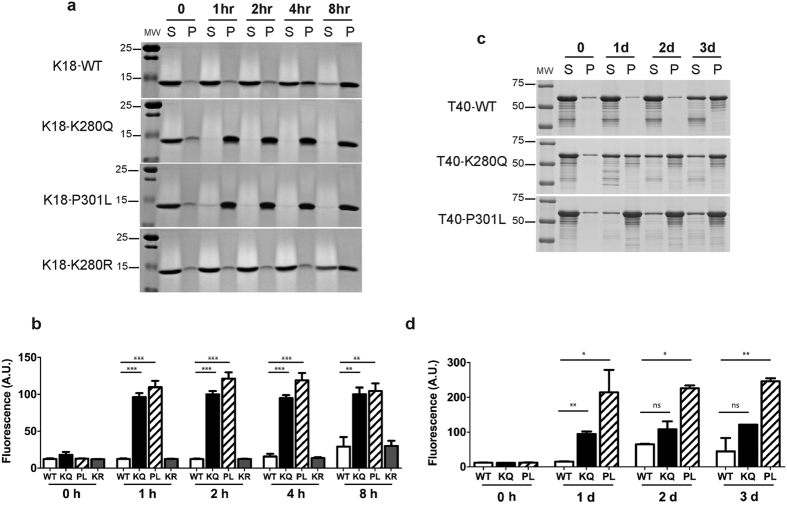
Tau K280 acetylation-mimic enhances tau aggregation *in vitro*. (**a**) Coomassie blue staining of monomeric supernatant (S) and fibrillar pellet (P) fractions of tau-K18 fibril reactions (0 to 8 h). **(b**) Thioflavin T (ThT) fluorescence of tau-K18 fibril reactions at the indicated time points from 0 to 8 h. Error bars indicate s.d of the mean. **(c**) Coomassie blue staining of monomeric supernatant (S) and fibrillar pellet (P) fractions of full-length tau-T40 fibril reactions (0 to 3 d). **(d**) ThT fluorescence of tau-T40 fibril reactions at the indicated time points from 0 to 3 d. Error bars indicate s.d of the mean derived from N = 3 independent experiments. Cropped images from full size gels are provided in panels a and c. Full-length gels are presented in Supplementary Fig. S4. Error bars indicate s.d. of the mean. Statistical significance was assessed using a student t-test (*p < 0.05; **p < 0.01; ***p < 0.001).

Given that acetylated tau is detected within NFTs in AD brain^4^,^11^,^12^, we next investigated whether acetylation might promote tau aggregation, as our previous study using CBP-acetylated tau suggested that fully acetylated tau induced tau aggregation *in vitro*^4^, however, the critical residues mediating this effect remained unclear. *In vitro* heparin-induced sedimentation assays were performed using acetylation-mimic and non-mimic tau proteins as well as the aggregate-prone familial P301L mutant. WT and K280R non-mimic tau-K18 proteins transitioned to the pellet fraction by 8 h. Strikingly, the K280Q mutant showed remarkably accelerated sedimentation kinetics and was near ~100% pelleted by 1 hr, an effect that was similarly observed with the positive control P301L mutant (Fig. 3a, Supplementary Fig. S4). Consistent with the sedimentation results, ThT fluorescence measurements detected robust tau aggregation that was ~5–6 fold higher for K280Q and P301L proteins at 1 h and remained elevated for the duration of the 8 h incubation period (Fig. 3b). The single K280Q and double K280/K281Q mutants showed comparable *in vitro* aggregation kinetics within this accelerated 0–1 h time-frame (data not shown). These results were further evaluated using full-length tau-T40 proteins containing single K280Q acetylation-mimic or P301L mutations. We observed enhanced transition to the pellet fraction for T40-K280Q and T40-P301L by 1 d (Fig. 3c, Supplementary Fig. S4), which was further corroborated by ThT fluorescence (Fig. 3d). Thus, K → Q substitutions within the critical PHF6* motif, in the apparent absence of other tau PTMs, appears sufficient to accelerate tau aggregation *in vitro*.

**Figure 4:**
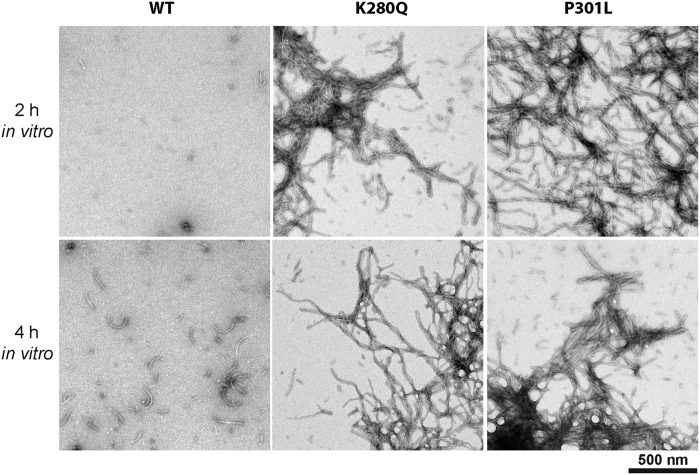
Tau K280 acetylation-mimic fibrils show similar ultrastructural properties to P301L mutant tau. Negative staining electron microscopy (EM) of tau-K18 protein fibril reactions incubated for 2 h (top row) and 4 h (bottom row). Scale bar, 500 nm.

Negative staining electron microscopy (EM) was performed to visualize fibril formation of WT, K280Q, and P301L tau-K18 at various time-points (Fig. 4). After 2 h incubation, only sparse WT fibrils were detected, in agreement with slower kinetic rates of aggregation. In contrast, numerous dense networks of mutant K280Q and P301L fibrils were observed after 2 h incubation, consistent with the accelerated sedimentation and ThT analysis for these two mutants. Furthermore, even after 4 h incubation, WT fibrils were not abundant, while the K280Q and P301L mutants continued to accumulate robust negatively stained filaments (Fig. 4). We were unable to detect robust K280R fibrils at any early time points, consistent with delayed aggregation kinetics.

**Figure 5:**
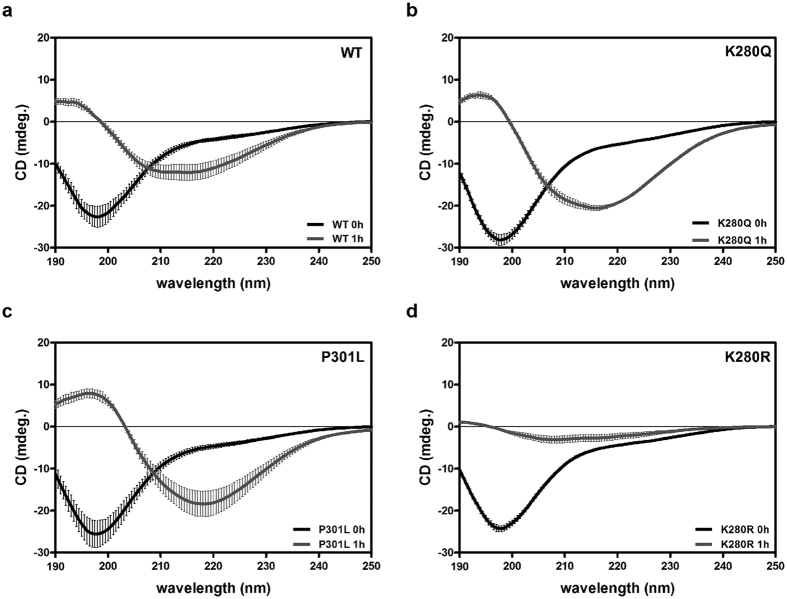
Tau K280 acetylation-mimic accelerates the formation of β-structure. (**a–d**) CD spectra of tau-K18 fibril reactions at the indicated time points from 0 to 1 h (0 h, black line; 1 h, grey line) were recorded from 190 to 250 nm. 0 h time points indicate monomeric soluble tau (random coil) and 1 h time point indicates fibrillar tau derived from the pellet fraction. Error bars indicate standard deviation (s.d.) of the mean derived from N = 3 independent experiments.

Since tau K280 acetylation is associated with amyloid tau pathology, we sought to further characterize the biochemical properties of aggregate-prone K280Q fibrils. Circular dichroism (CD) was employed to assess whether K280Q fibrils contained anti-parallel β-sheet structure, which strongly correlates with tau aggregation. As shown in Fig. 5, K280Q and P301L fibrils incubated for only 1 h showed the strongest shift in absorbance from random coil structure to β-sheet structure, as determined by the peak broadening observed at ~218 nm, a conformational transition previously noted for aggregate-prone tau fibrils and indicative of tau amyloid structure *in vivo*^21^,^22^. While WT fibrils displayed some detectable β-structure at the 1 h time-point, we note that the amyloid conversion for both WT and, more prominently, the K280R fibrils was reduced when compared to aggregate-prone K280Q or P301L mutants, also consistent with their delayed aggregation kinetics.

**Figure 6:**
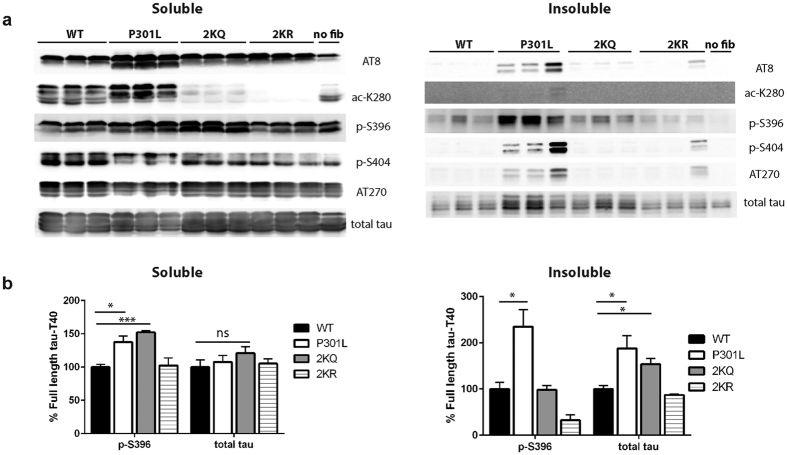
Tau acetylation-mimics enhance seed-dependent tau aggregation in cells. (**a**) Total and phospho-tau immunoblotting of full-length tau-T40 expressing QBI-293 cells (WT, P301L, 2KQ, 2KR) seeded with K18-PL fibrils or, as a negative control, no fibril treatment (no fib). (**b**) Quantification of immunoblots in **(a)** was performed by protein band densitometry. Cropped images from full size immunoblots are provided in panel a. Full-length immunoblots and additional quantification are presented in Supplementary Fig. S5. Statistical significance was assessed using a student t-test (*p < 0.05; ***p < 0.001).

Fibrillar tau seeds promote the rapid appearance of K280-acetylated tau in mice^13^,^23^. We therefore tested whether expression of full-length tau containing acetylation-mimics, T40-2KQ (K280/281Q), was susceptible to seed-dependent tau aggregation in a cell-based assay. K18-P301L seeds were packaged and delivered into cultured QBI-293 cells ectopically expressing full-length tau-T40 (WT, P301L, 2KQ, or 2KR) and seeded tau pathology was assessed by immunoblotting of soluble and insoluble fractions. As expected, full-length T40-P301L partly accumulated in the insoluble fraction and was detectable with a panel of phospho-tau and acetyl-tau (ac-K280) antibodies (Fig. 6a and b, Supplementary Fig. S5). Similar to T40-P301L, full-length T40-2KQ also showed increased aggregation upon seeding and accumulated in the insoluble fraction, as detected with a total tau antibody (K9JA). In contrast, the T40-2KR non-mimic mutant was not significantly aggregated and behaved similarly to WT. Interestingly, unlike T40-P301L, which was hyper-phosphorylated at all epitopes examined, the insoluble T40-2KQ pool did not show robust phospho-tau immunoreactivity, indicating that tau hyper-phosphorylation does not necessarily correlate with insoluble tau seeding (Fig. 6b and Supplementary Fig. S5). Thus, acetylation-mimic tau mutations at residues K280/K281 are capable of enhancing the seed-dependent aggregation of full-length tau.

### Cysteine oxidizing compounds modulate acetylation-induced tau aggregation

**Figure 7:**
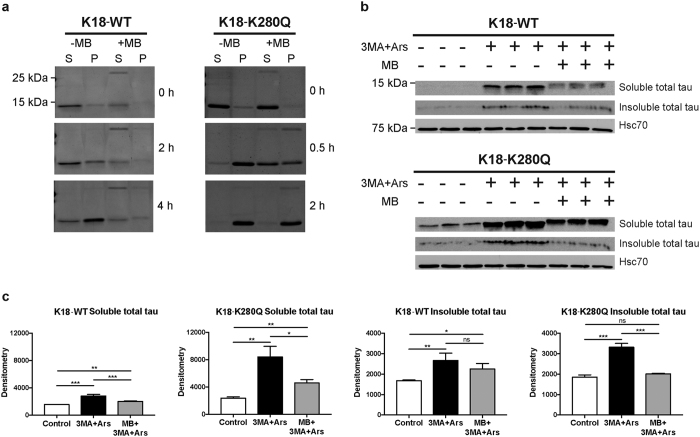
Methylene blue (MB) suppresses the accumulation of tau K280 acetylation-mimic. (**a**) Coomassie blue staining of monomeric supernatant (S) and fibrillar pellet (P) fractions of tau-K18 WT and K280Q fibril reactions in the absence of presence of MB. (**b**) Immunoblot analysis with indicated antibodies of cells expressing K18-WT or K18-K280Q treated with 3MA, Ars, and/or MB, where indicated. Hsc70 was included as a loading control. (**c**) Quantification of immunoblots in (**b**). Error bars indicate s.d. of the mean. Statistical significance was assessed using a student t-test (*p < 0.05; **p < 0.01; ***p < 0.001). Cropped images from full size gels and immunblots are provided in panels a and b. Full-length gels and immunoblots are presented in Supplementary Fig. S6.

Given that the acetylation-mimics enhanced tau aggregation, we searched for compounds that might inhibit this process. Methylene blue (MB) and its derivatives are a class of anti-tau aggregation compounds, but their mechanism of action is not fully understood^24^,^25^,^26^,^27^,^28^,^29^,^30^,^31^. We tested whether MB inhibited WT and K280Q tau aggregation *in vitro*. Tau proteins were pre-incubated with MB prior to the initiation of fibril reactions. Consistent with previous reports, MB inhibited WT tau-K18 fibrillization as indicated by the delayed appearance of ~15 kDa tau-K18 in the fibrillar pellet fraction (Fig. 7a, left, Supplementary Fig. S6)^26^. Although K280Q showed accelerated fibril formation at 30 min, MB pre-treatment was also sufficient to partially delay K280Q aggregation at this time point, but was much less effective at 2 h, a time point at which K280Q had more fully transitioned to the fibrillar pellet fraction (Fig. 7a, right, Supplementary Fig. S6). The appearance of a ~30 kDa tau dimer was also observed in the presence of MB, consistent with its reported cysteine oxidizing properties^26^.

To assess the effects of MB on tau in cultured cells, we employed a previously described pharmacological strategy combining autophagy inhibition using 3-methyladenine (3MA) and exposure to the oxidative stressor sodium arsenite (Ars), which together stabilize the accumulation of aberrantly modified tau^10^. K18-WT, and more prominently K18-K280Q, accumulated in the soluble and also partially in the insoluble fractions in response to 3MA and Ars, and their levels were suppressed by co-incubation with MB (Fig. 7b and c, Supplementary Fig. S6). Further implicating aberrant tau PTMs in this process, K18-P301L expressing cells showed increased K280 acetylation and S262 phosphorylation within the MTBR, and these were also suppressed by co-incubation with MB (Supplementary Fig. S6). Thus, MB is sufficient to reduce aberrantly modified tau species in a cell-based assay, although we note that MB has broad pleiotropic effects in the cell that could also influence tau including regulation of tau kinases and caspases^32^,^33^.

Given the absence of ectopically expressed acetyltransferases in this cell-based assay, we hypothesized that MB may act, in part, by chemically interfering with endogenous tau acetylation, thereby decreasing aberrantly acetylated tau species and preventing their accumulation. Tau auto-acetylation was recently reported to occur in a cysteine-dependent manner in the complete absence of CBP/p300, in which acetyl-CoA reactive tau cysteines facilitate intrinsic tau acetylation on lysines^7^,^10^,^34^. Since MB is known to interact with tau cysteines^24^,^26^, we tested whether MB inhibits tau auto-acetylation using an *in vitro* radiolabeling assay (Supplementary Fig. S6). Inhibition of tau auto-acetylation was not observed with inactive control compounds. However, pre-incubation of K18-P301L with either MB or aminothienopyridazines (ATPZs), both of which are proposed to bind tau cysteines and promote their oxidation^26^, was sufficient to partially inhibit tau auto-acetylation (Supplementary Fig. S6). These results suggest that MB and related cysteine-interacting compounds could act, in part, via inhibition of tau auto-acetylation activity.

## Discussion

In this study, we provide evidence that acetylation at residues K280/K281 is critical for normal and pathological tau functions. Site-specific tau acetylation was sufficient to alter the tau post-translational profile, impair tau-mediated MT stability, and accelerate tau aggregation. Furthermore, we provide evidence that acetylated full-length tau is more susceptible to seed-dependent tau aggregation, implicating tau acetylation in the pathogenic cascade leading to tau pathology. These results warrant future efforts to target tau acetylation as a means to restore the stabilization of MTs, a process that is perturbed during the progression of AD and related tauopathies.

The identification of K280-acetylated tau in tauopathy brains initially presented a paradox regarding the significance of this residue in neurodegeneration. How can both acetylation of K280, detected in AD and related 4R-tauopathies, and the genetic deletion of the same residue, ΔK280 seen in FTDP-17 patients, both be mechanistically linked to tauopathy? Given the critical role for residues K280/K281 in mediating MT interactions^17^,^35^,^36^,^37^,^38^, we propose that either acetylation-induced charge neutralization or complete deletion of lysines altogether yield comparable phenotypic consequences in reducing the positive charge and therefore the affinity of tau for MTs, thereby facilitating the accumulation of cytosolic tau aggregates. Indeed, either K → Q substitutions, expression of the acetyltransferase CBP/p300, or deletion of K280/K281 altogether (i.e. ΔK280/281) led to similar compensatory tau dephosphorylation (Fig. 1) and loss of MT-stabilizing functions (Fig. 2). Thus, it is intriguing that a lysine PTM phenocopies a genetic lesion (e.g. ΔK280), providing a plausible explanation for how WT tau could become aggregated in the vast majority of sporadic tauopathies including AD. In fact, all 4R-tauopathies analyzed to date have shown some degree of tau K280 acetylation, which correlated most significantly with Thioflavin-positive mature NFTs in AD brains^11^,^12^. Importantly, however, unlike genetic tau mutations, abnormal tau PTMs such as acetylation provide a modifiable target for therapeutic intervention. Therefore, enhancement of deacetylase activity (e.g. HDAC6 or SIRT1)^4^,^8^,^39^ or inhibition of acetyltransferase activity (e.g. CBP/p300 or auto-acetyltransferase activity)^4^,^8^,^10^,^15^ could potentially alleviate tau aggregation and enhance normal tau functions. In this regard, the recent identification of salsalate as CBP/p300 inhibitor targeting tau K174 acetylation may also be effective at reducing K280 acetylation levels^15^. Future studies could evaluate whether any known or emerging CBP/p300 inhibitors modulate K280/K281 acetylation.

In addition to CBP/p300, which is predominantly a nuclear enzyme involved in transcription and gene expression, our previous work suggested an intrinsic tau auto-acetylation activity capable of mediating tau self-acetylation via acetyl-CoA reactive cysteine residues^10^,^34^,^40^. Although the extent of tau auto-acetylation that occurs *in vivo* is not known, we speculate that a dedicated tau acetyltransferase may not be necessary to sustain increased tau acetylation in AD brain. Recent studies support the notion that physiological concentrations of acetyl-CoA cofactor alone could facilitate acetylation of cytoplasmic substrates^41^,^42^,^43^. Our results *in vitro* and in cell-based assays showed that MB or ATPZs, compounds that reportedly promote cysteine oxidation and prevent tau aggregation, may also impair tau auto-acetylation. While we cannot rule out the contribution of other yet-to-be-identified tau acetyltransferases, our study suggests that cysteine oxidizing compounds might directly interfere with tau’s intrinsic cysteine-dependent acetyl group transfer, thereby modulating tau aggregation. Therefore, we speculate that any neuroprotective effects of MB and/or its derivatives could arise, in part, from their inhibition of tau acetylation *in vivo*. If so, future drug discovery efforts to identify compounds that selectively target tau cysteines may represent viable approaches to prevent tau acetylation and limit the accumulation of tau pathology.

Given the complex post-translational regulation of tau by a variety of PTMs, we investigated potential cross-talk between acetylated tau and known pathological phospho-tau epitopes. Surprisingly, we found that tau acetylation led to its dephosphorylation at the AT8 epitope via PP2A phosphatase activity (Fig. 1). Elevated phosphatase activity likely acts in a compensatory manner to re-initiate tau-MT binding and/or prevent phospho-tau accumulation. The acetylation-induced dephosphorylation at AT8 is consistent with previous reports that AT8 is a high affinity and preferred PP2A target site^44^,^45^,^46^. Interestingly, a transient reduction in AT8 immunoreactivity has been noted in a tau transgenic animal model^47^, indicating that fluctuations and/or dephosphorylation at the AT8 epitope could represent an intermediate step prior to subsequent pathological modifications. Since PP2A activity is impaired with AD progression^48^,^49^, AT8 might eventually re-emerge at later stages of disease progression characterized by tau hyper-phosphorylation and aggregation. Supporting this possibility, transient AT8 reduction and recovery has been observed in cell culture models challenged with oxidative or cytoskeletal damaging agents^50^,^51^. More recently, phosphorylation of one residue comprising the AT8 epitope (T205) was shown to be neuroprotective in response to excitotoxicity, suggesting that acetylation could promote toxicity by reducing T205 phosphorylation^52^. The complex relationship between tau acetylation and phosphorylation highlights an incomplete understanding of dynamic tau PTMs, and therefore global tau PTM analysis, as opposed to specific tau epitopes of interest, may provide more accurate predictors of tau pathogenesis.

Two prior observations hint at an involvement of tau acetylation in prion-like tau propagation. First, in both cell-based^8^ and tau transgenic mouse models^4^, increased Aβ levels were sufficient to induce tau acetylation, which could facilitate tau spreading into higher neocortical brain regions. Secondly, injection of recombinant tau fibrils into the hippocampus of PS19 mice led to intracellular tau acetylation within two weeks post-injection of fibrils^13^, an unexpected observation that may be linked to subsequent tau propagation *in vivo*. Here, full-length tau containing K280/K281 acetylation-mimics (T40-2KQ) showed enhanced seed-dependent tau aggregation (Fig. 6). Since tau proteins are highly acetylated in AD brain, but not mutated in AD, it is conceivable that K280/K281 acetylated tau may better reflect the pathogenic tau species capable of initial aggregation and subsequent propagation in a spatiotemporal manner through the brain. More detailed biochemical analysis of the PTMs present on tau oligomers or other potentially transmissible tau species are necessary to further establish a causal link between acetylation and tau propagation.

In summary, our study highlights tau acetylation as a critical pathogenic mechanism that may confer both loss and gain of function toxicity in AD and related tauopathies. Given the central location of acetylated lysine clusters within the MTBR, we propose that acetylation, particularly at residues K280/K281 as well as other lysines that physically engage MTs, mediates loss of MT-regulatory function and gain of abnormal tau aggregation in sporadic tauopathies including AD. Since tau contains many lysines that are subject to acetylation, future approaches that merge genetic, biochemical, neuropathological, and physiological techniques will collectively be required to dissect this complex tau PTM code. These insights are essential to devise new therapeutic strategies designed to selectively modify specific tau PTMs in AD patients.

## Methods

### Plasmids and Cell Culture

Two versions of human tau were used in this study. Most experiments used the full-length tau isoform containing both N-terminal inserts and all four repeat domains (2N4R-tau also designated tau-T40). The impact of MB and ATPZs used a fragment of tau comprising only the four repeat domains (designated tau-K18). All tau-T40 and tau-K18 plasmids were cloned into the pCDNA5/TO vector (Life Technologies). The ΔKK, K → Q, K → R, and P → L tau mutations were created using site-directed mutagenesis (NEB). In the tau-T40 and enzyme co-transfection experiments, the following enzyme plasmids were used: CBP WT, CBP LD, (CBP L1435A/D1436A), and HDAC6 (generous gifts from Tso-Pang Yao, Duke University, Durham, NC); GSK3 (generous gift from Virginia M.-Y. Lee, University of Pennsylvania, Philadelphia, PA); and PP2A (PP2A-B56 α subunit, Addgene plasmid #14532). QBI-293 cells were grown in full DMEM media (supplemented with 10% FBS, 1X L-glutamine, and 1X penicillin/streptomycin), and FuGENE 6 transfection reagent (Promega) was used to perform the QBI-293 cell transfections.

### Okadaic Acid (OA) Treatment Experiments

For OA treatment, QBI-293 cells transfected with tau-T40 plasmids were treated with 25 nM OA for 16 h. Cells were lysed in RIPA buffer [50 mM Tris pH 8.0, 150 mM NaCl, 5 mM EDTA, 0.5% sodium deoxycholate, 1% Igepal CA-630, 0.1% SDS] supplemented with deacetylase, phosphatase and protease inhibitors as follows: 2 μM Trichostatin A (TSA), 10 mM Nicotinamide (NCA), 1 mM sodium fluoride (NaF); 1 mM sodium orthovanadate (Na_3_VO_4_); 1 mM phenylmethylsulfonyl fluoride (PMSF); and protease inhibitor cocktail composed of L-1-tosylamide-2-phenylethylchloromethyl, *N*-tosyl-L-lysine chloromethyl ketone, leupeptin, pepstatin, and soybean trypsin inhibitor, each at 1 μg/ml). The samples were sonicated 20 times and centrifuged at 15,000 rpm for 30 min at 4 °C and supernatants were resolved by sodium dodecyl sulfate**-**polyacrylamide gel electrophoresis (SDS-PAGE).

### Neuronal Transfection and Fluorescent Microscopy

All animal studies were carried out in accordance with the University of North Carolina (UNC) Institutional Animal Care and Use Committee (IACUC). The animal protocol was approved by the UNC IACUC Committee (ID# Number: 14.107.0). Primary cortical neurons were cultured from E16 CD1 mouse embryos (Charles River) and were plated onto poly-D-lysine coated coverslips. Neuron transfection of 1 μg of tau-GFP pCDNA was performed using CalPhos™ Mammalian Transfection Kit in accordance with manufacturer instructions (Clontech). Fixation was accomplished by incubating the coverslips with 4% paraformaldehyde for 15 min, and cells were permeabilized by exposing coverslips to a 0.1% Triton X-100 in 1X PBS solution for 8 min to permeabilize the cells. Blocking was performed for 1 h at least an hour at room temperature in a solution of 2% milk in TBS containing 0.5% Triton. Following blocking, coverslips were then incubated in primary antibody, AT8 (1:1000), overnight at 4 °C. This was followed by staining with an Alexa Fluor^®^ 594-conjugated secondary antibody and counterstaining with DAPI.

### Mass spectrometry

NanoLC nanospray MS-MS analysis was performed at the University of Pennsylvania proteomics core facility, as previously described^4^. Briefly, QBI-293 cells were co-transfected with tau-T40 WT and CBP and tau was immunoprecipitated with T14/T46 antibodies. After separating tau via SDS-PAGE, gel bands were excised and submitted for mass spectrometry analysis using LTQ XL* Linear Ion Trap Mass Spectrometer (Thermo Scientific). Data was acquired using Xcalibur software (Thermo Scientific) and analyzed using Mascot, Scaffold, and PEAKS software programs. Unambiguously acetylated peptides were determined by significant 10logP scores >30 and mass accuracy scores <1.0 ppm.

### Protein Expression and Purification

Protein expression, extraction, and purification was performed using chromatography methodology to purify heat stable tau proteins. Tau-K18 and tau-T40 plasmids were cloned into the pRK172 bacterial expression vector for inducible protein expression. Protein was expressed in BL21 (DE3) RIL *E. coli* cells. Bacteria were grown in lysogeny broth, ampicillin was added, and when an OD of 1.0 was reached, protein expression was induced with isopropyl-β-D-thiogalactopyranoside at a final concentration of 1.0 mM. After continued growth for 2 h, bacterial cultures were then centrifuged at 5,000 rpm for 15 min, and pellets were immediately frozen at −80 °C. Pellets were resuspended in a high salt RAB buffer, pH 7.0 (0.1 M MES, 1 mM EGTA, 0.5 mM MgSO_4_, 750 mM NaCl, 20 mM NaF, 0.1 mM PMSF, 0.1% protease inhibitor cocktail). This resuspension was homogenized, boiled, and centrifuged at 100,000 × g for 30 min. The resulting supernatant, with addition of 0.1 mM PMSF and 0.1% protease inhibitor cocktail, was dialyzed against FPLC buffer, pH 6.5 [20 mM piperazine-N,N’-bis(ethanesulfonic acid), 10 mM NaCl, 1 mM EGTA, 1 mM MgSO_4_, 0.1 mM PMSF and 2 mM dithiothreitol (DTT)]. After overnight dialysis, the contents were passed through a HiTrap sulfopropyl sepharose high performance cation exchange column (GE) attached to an ÄKTA Pure chromatography system equilibrated in FLPC buffer. Fractions were eluted over a 0–0.4 M NaCl gradient. Portions of the fractions were separated by SDS-PAGE and stained with Coomassie Blue R-250. Fractions containing tau protein were subsequently pooled. The FPLC buffer present in pooled tau protein fractions was exchanged for 100 mM sodium acetate (pH 7.0), and protein was concentrated using Amicon Ultra centrifugal filter devices (Millipore Corporation, Billerica, MA). Resultant protein concentration was determined using bicinchoninic acid assay (Thermo Scientific Pierce).

### Tau Fibrillization Reactions

For sedimentation analysis, Thioflavin T, and negative stain electron microscopy, a concentration of 10 μM tau-K18 was incubated without agitation at 37 °C with 10 μM heparin (Sigma) and 2 mM DTT in a 100 mM sodium acetate buffer (pH 7.0). In the MB sedimentation experiments, a fibril reaction mixture with 10 μM tau-K18 and 50 μM MB (Sigma) was held on ice for 15 minutes prior to adding heparin to a final concentration of 10 μM. Tau-T40 incubations were performed in a similar fashion, but used 15 μM tau-T40/15 μM heparin and were agitated at 37 °C. Fibril reactions for circular dichroism and cell seeding assay used 20 μM tau-K18/20 μM heparin.

### Sedimentation Analysis by SDS-PAGE

Tau fibril reactions were centrifuged at 15,000 rpm for 30 min at 4 °C to separate supernatant and pellet fractions. The pellet fraction was resuspended in 1X loading buffer and 100 mM DTT and an equal mass of supernatant was mixed with 6X loading buffer and 100 mM DTT. Equal mass supernatant and pellet fractions for various reaction times were separated by SDS-PAGE followed by Coomassie blue staining to detect tau protein.

### Thioflavin T (ThT) Fluorescence Assay

The ThT signal of tau-K18 and tau-T40 fibril reactions at various reaction times was measured at 430 nm (excitation) and 500 nm (emission) in a 10 μM ThT solution using a FLUOstar Omega microplate reader (BMG LABTECH, Germany). Into each well, 15 µl of protein and 150 µl of 10 µM ThT solution was added. The plate was shaken briefly in the plate reader, and then the measurements were performed.

### Negative Staining Electron Microscopy (EM)

For EM experiments, 5 μL of 10 μM tau-K18 fibril reactions were placed on 400 mesh Formvar/Carbon film-coated copper grids (Ted Pella, Inc.) for 5 min, quickly washed with distilled water two times, and then stained with 2% uranyl acetate for 30 sec. A LEO EM910 transmission electron microscope (Carl Zeiss Microscopy) at an acceleration voltage of 80 kV was used to visualize the fibrils. The camera used was an Orius SC1000 CCD Camera, 2672 × 4008 pixels (11 megapixels) and Digital Micrograph version 2.3 software was used to acquire the images (Gatan, Inc.). A minimum of N = 10 independent regions of each grid was imaged from N = 3 independent replicates of WT, K280Q, or P301L mutant fibrils.

### Circular Dichroism (CD)

Measurements for CD were performed with a Chirascan^TM^ circular dichroism spectrometer (Applied Photophysics Ltd, United Kingdom) using a cuvette with a 0.10 cm path length. CD data was collected from 190 nm to 250 nm at 0.5 nm intervals. For the 0 h time point, 20 μM of the tau-K18 monomer in 100 mM sodium acetate buffer was dialyzed overnight against the CD buffer [10 mM potassium phosphate, 100 mM potassium fluoride pH 7.0]. For 1 h time point, CD spectra were generated using 20 μM tau fibril reaction pellet fractions resuspended in CD buffer.

### Tubulin Polymerization Assay

Tubulin polymerization at 37 °C was monitored via absorbance readings at 350 nm using a FLUOstar Omega microplate reader (BMG LABTECH, Germany). Polymerization reactions contained 2 mM GTP (Sigma), 55 μM purified tubulin (Cytoskeleton), and 40 μM purified tau-K18 protein (or no tau in case of control). The reactions were carried out in PEM buffer [80 mM piperazine-N,N’-bis(ethanesulfonic acid) pH 6.9, 0.5 mM EGTA, 2 mM MgCl_2_ hexahydrate]. Absorbance data was collected at 1 min intervals for 45 min.

### Methylene Blue (MB) Cell Treatment Assay

QBI-293 cells transfected with tau-K18 WT, K280Q, or P301L were treated with 10 μM MB before and after treatment with 10 mM 3-methyladenine (3MA) and 40 μM sodium arsenite (Ars). After overnight treatment with MB, 3MA and Ars, the cells were lysed on ice and sequential extraction was performed. Cells were lysed in RIPA buffer [50 mM Tris pH 8.0, 150 mM NaCl, 5 mM EDTA, 0.5% sodium deoxycholate, 1% Igepal CA-630, 0.1% SDS] supplemented with deacetylase, phosphatase and protease inhibitors (2 μM TSA, 10 mM NCA, 1 mM NaF; 1 mM Na_3_VO_4_; 1 mM PMSF; and protease inhibitor cocktail). The samples were sonicated 20 times and centrifuged at 15,000 rpm for 30 min at 4 °C. Another extraction was performed on the pellet fraction using urea buffer [7 M urea, 2 M thiourea, 4% CHAPS, 30 mM Tris pH 8.5] supplemented with the same deacetylase, phosphatase, and protease inhibitors as the RIPA buffer. The samples were sonicated 10 times and centrifuged at 15,000 rpm for 30 min at room temperature. For radiolabeling experiments, total tau proteins (1 μg total protein) were acetylated by incubation in 30 μl of reaction buffer (50 mM Tris-HCl pH 8.0, 10% glycerol, 1 mM DTT, 100 mM EDTA, 1 mM PMSF, and 10 nCi [^14^C]-labeled acetyl-CoA acetyl-CoA) for 1 h at 37 °C. Acetylation reactions were pre-incubated for 10 min with 20 μM MB or active/inactive ATPZ compounds, which were kindly provided by Dr. Kurt Brunden (University of Pennsylvania).

### Tau-K18 Fibril Seeding Assay

QBI-293 cells transfected with indicated tau-T40 plasmids were transduced with tau-K18-PL fibrils or no fibril control. Each fibril treated well of the 6-well plate received 6 μg of tau-K18-PL fibrils. Fibrils treatments were prepared by resuspending pellets from tau-K18-PL fibril reactions in OptiMEM and then sonicating this mixture 30 times. No fibril controls were OptiMEM alone. The sonicated fibrils (or no fibril controls) were then added to OptiMEM plus Lipofectamine2000 (Invitrogen) and allowed to stand for 20 min at room temperature. This fibril-Lipofectamine mixture was added to the transfected cells. After an overnight incubation, cell culture media was replaced with fresh, full DMEM media. Cells were lysed 44–48 h after fibril addition. They were harvested on ice into Triton X-100 lysis buffer [1% Triton X-100, 150 mM NaCl, 50 mM Tris pH 7.6] supplemented with deacetylase, phosphatase and protease inhibitors [3 μM TSA, 10 mM NCA, 1 mM NaF; 1 mM Na_3_VO_4_; 1 mM PMSF; and protease inhibitor cocktail]. The samples were sonicated 30 times and centrifuged at 15,000 rpm for 30 min at 4 °C. The supernatant was reserved as the Triton-soluble fraction. A second Triton X-100 extraction was performed on the pellet using the same parameters as the first Triton X-100 extraction. The last extraction of the pellet was performed using SDS lysis buffer [1% SDS, 150 mM NaCl, 50 mM Tris pH 7.6] supplemented with the same deacetylase, phosphatase, and protease inhibitors as the Triton X-100 lysis buffer. The samples were sonicated 30 times and centrifuged at 15,000 rpm for 30 min at room temperature. The resulting supernatant was reserved as the Triton-insoluble SDS fraction.

### Immunoblotting and antibodies

Cell lysates were resolved via SDS-PAGE (on 10% gels for tau-T40 and 15% gels for tau-K18), transferred to nitrocellulose membrane (Biorad), and blocked with 2% milk in 1X TBS for 30 min. Membranes were incubated with primary antibody overnight at 4 °C, followed by a 1 h room temperature incubation with HRP-conjugated anti-mouse or anti-rabbit antibodies (1:1000). The following primary antibodies were used: pSer202/pThr205-tau (AT8, 1:1000, Thermo Fisher Scientific, MN1020), pSer262-tau (p-S262, 1:1000, Thermo Fisher Scientific, 44-750G), pThr231-tau (AT180, 1:1000, Thermo Fisher Scientific, MN1040), pThr181-tau (AT270, 1:1000, Thermo Fisher Scientific, MN1050), tau-1 (1:1000, Millipore, MAB3420), pSer396-tau (p-S396, Thermo Fisher Scientific, 44-752G), anti-acetylated K280-tau^4^ (ac-K280, 1:1000), tau K9JA (total tau, 1:5000, Dako, A0024), tau-T46 (1:1000, Thermo Fisher Scientific, 13-6400), and Hsc70 (1:1000, Enzo Life Sciences, SPA-815). ImageJ software (NIH) was used to quantify band intensity.

### Statistical analysis

Comparison between two groups were analyzed using a student t-test. Significance is presented with *, **, and ***corresponding to p < 0.05, p < 0.01, and p < 0.001, respectively. Error bars represent the SD of the mean. Statistical analysis was performed using GraphPad Prism software.

## Additional Information

**How to cite this article**: Trzeciakiewicz, H. *et al*. A Dual Pathogenic Mechanism Links Tau Acetylation to Sporadic Tauopathy. *Sci. Rep.* **7**, 44102; doi: 10.1038/srep44102 (2017).

**Publisher's note:** Springer Nature remains neutral with regard to jurisdictional claims in published maps and institutional affiliations.

## Supplementary Material

Supplementary Information
