# Supplementary material for: A Dual Pathogenic Mechanism Links Tau Acetylation to Sporadic Tauopathy
Source: Sci Rep. 2017 Mar 13;7:44102. doi: 10.1038/srep44102 (PMC5347034; doi:10.1038/srep44102)
Supplement: Supplementary Information [file srep44102-s1.pdf]

# A Dual Pathogenic Mechanism Links Tau Acetylation to Sporadic Tauopathy

Hanna Trzeciakiewicz, Jui-Heng Tseng, Connor M. Wander, Victoria Madden,
Ashutosh Tripathy, Chao-Xing Yuan, and Todd J. Cohen

Supplementary Information (Figures S1-S6)

Trzeciakiewicz et al., Supplementary Figure S1

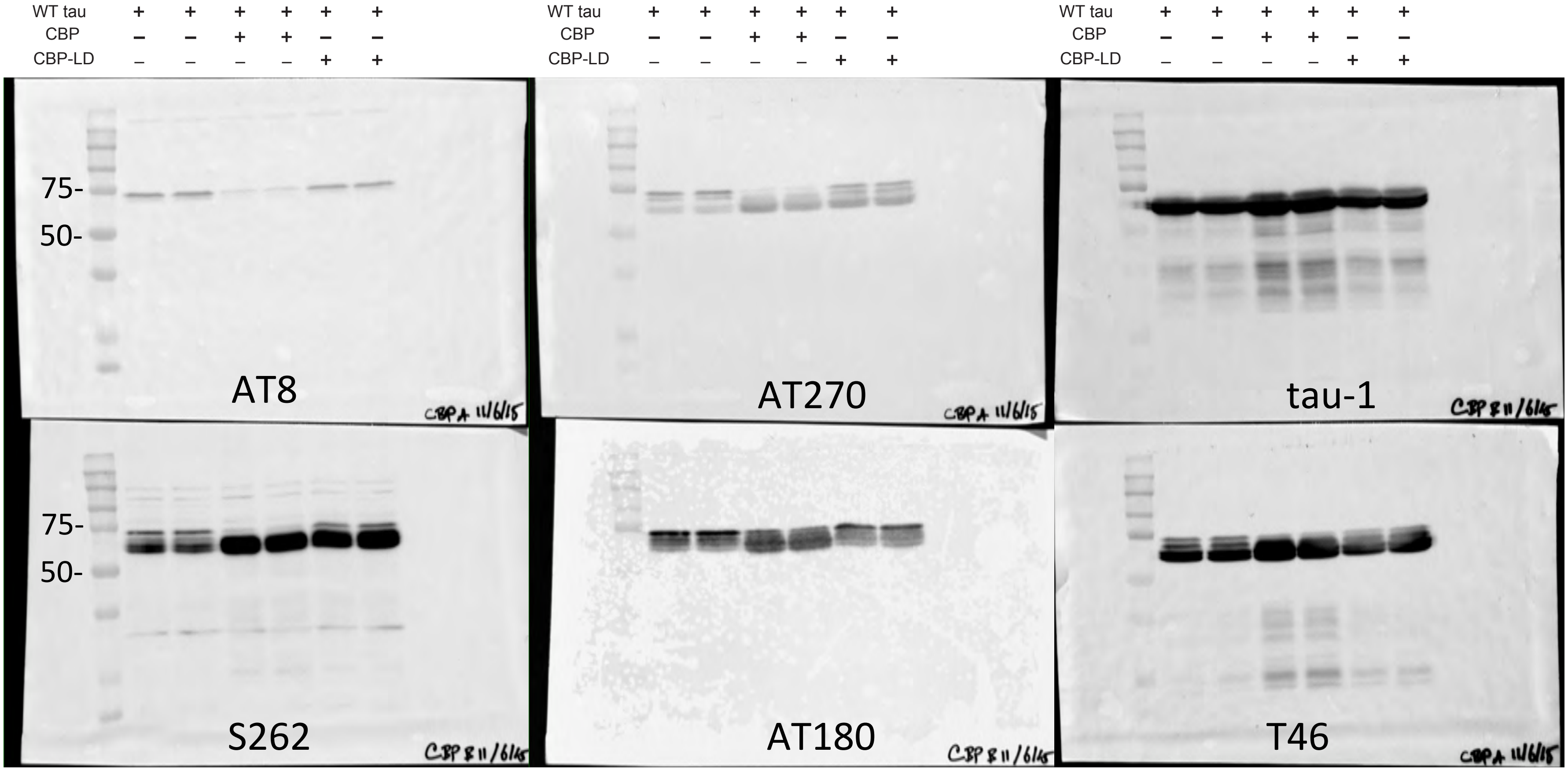


Full-length blots from Fig. 1a

Trzeciakiewicz et al., Supplementary Figure S1

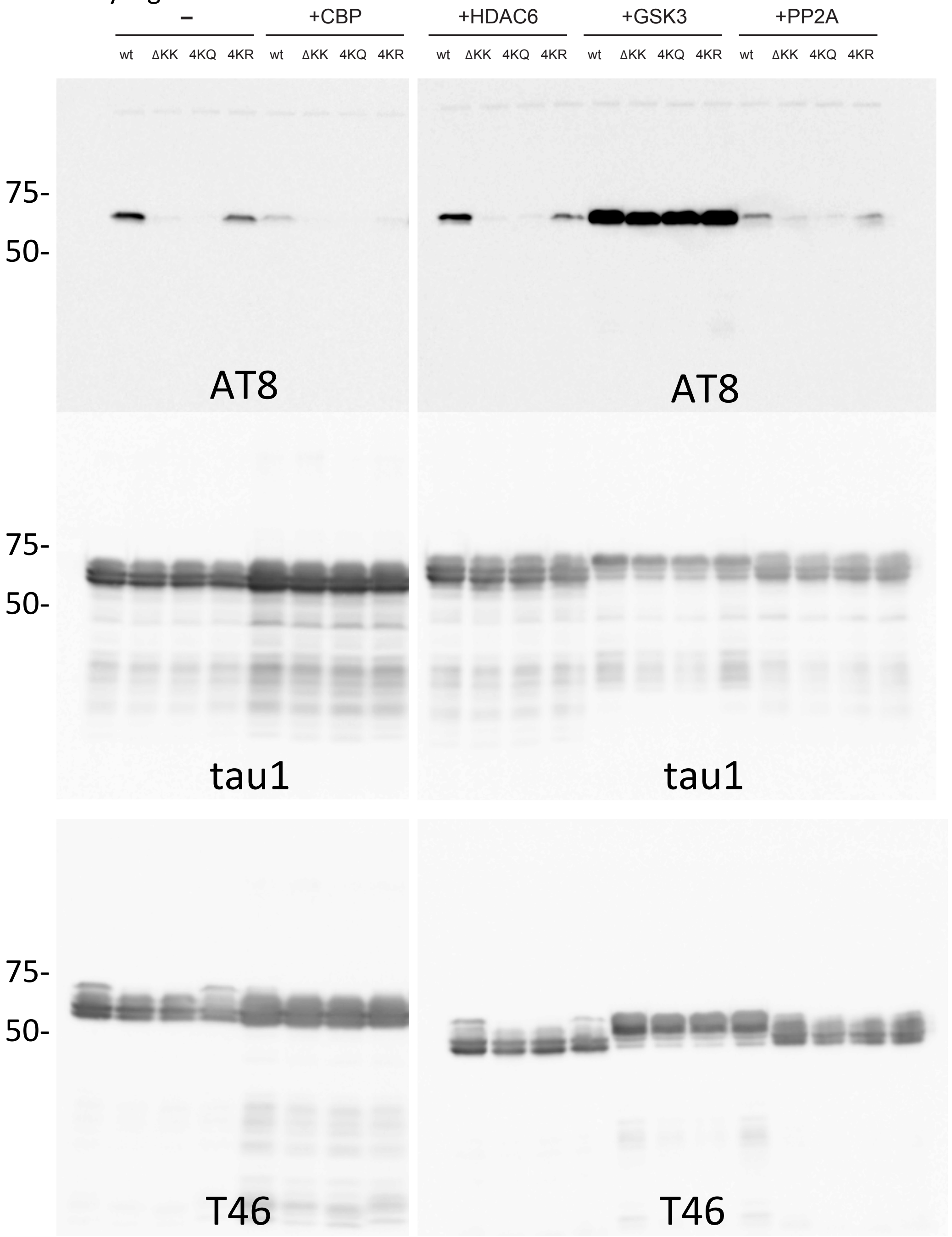


Full-length blots from Fig. 1b

Trzeciakiewicz et al., Supplementary Figure S1

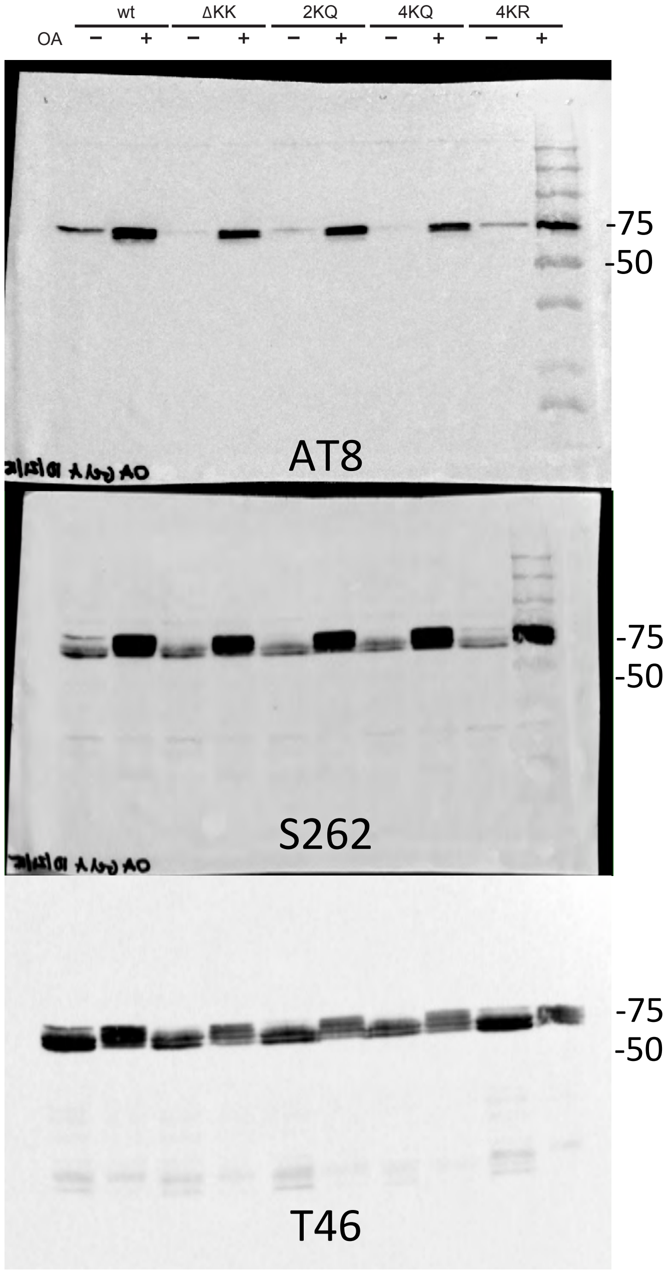


Full-length blots from Fig. 1c

| # | b | b-H2O | b-NH3 | b (2+) | Seq | y | y-H2O | y-NH3 | y (2+) | # |
|---|---|---|---|---|---|---|---|---|---|---|
| 1 | 100.08 | 82.07 | 83.05 | 50.54 | V | | | | | 16 |
| 2 | 228.13 | 210.12 | 211.11 | 114.57 | Q | 1812.02 | 1794.01 | 1795.00 | 906.51 | 15 |
| 3 | 341.16 | 323.21 | 324.19 | 171.11 | I | 1684.01 | 1665.95 | 1667.10 | 842.48 | 14 |
| 4 | 454.25 | 436.29 | 437.28 | 227.65 | I | 1570.85 | 1552.87 | 1553.87 | 785.94 | 13 |
| 5 | 568.31 | 550.46 | 551.27 | 284.67 | N | 1457.81 | 1439.86 | 1440.77 | 729.40 | 12 |
| 6 | 738.34 | 720.02 | 721.37 | 369.73 | K(+42.01) | 1343.86 | 1325.82 | 1326.73 | 672.38 | 11 |
| 7 | 908.62 | 890.47 | 891.53 | 454.78 | K(+42.01) | 1173.67 | 1155.72 | 1156.62 | 587.32 | 10 |
| 8 | 1021.68 | 1003.52 | 1004.61 | 511.32 | L | 1003.52 | 985.61 | 986.51 | 502.27 | 9 |
| 9 | 1136.75 | 1118.45 | 1119.64 | 568.83 | D | 890.47 | 872.57 | 873.43 | 445.73 | 8 |
| 10 | 1249.81 | 1231.93 | 1232.79 | 625.38 | L | 775.45 | 757.50 | 758.40 | 388.22 | 7 |
| 11 | 1336.84 | 1318.75 | 1319.76 | 668.89 | S | 662.35 | 644.41 | 645.32 | 331.67 | 6 |
| 12 | 1450.91 | 1433.01 | 1433.80 | 725.91 | N | 575.30 | 557.36 | 558.23 | 288.16 | 5 |
| 13 | 1549.95 | 1532.01 | 1532.87 | 775.45 | V | 461.27 | 443.37 | 444.24 | 231.14 | 4 |
| 14 | 1678.04 | 1660.15 | 1661.25 | 839.48 | Q | 362.15 | 344.14 | 345.19 | 181.60 | 3 |
| 15 | 1765.06 | 1747.14 | 1747.96 | 882.99 | S | 234.14 | 216.13 | 217.12 | 117.57 | 2 |
| 16 | | | | | K | 147.11 | 129.10 | 130.09 | 74.06 | 1 |

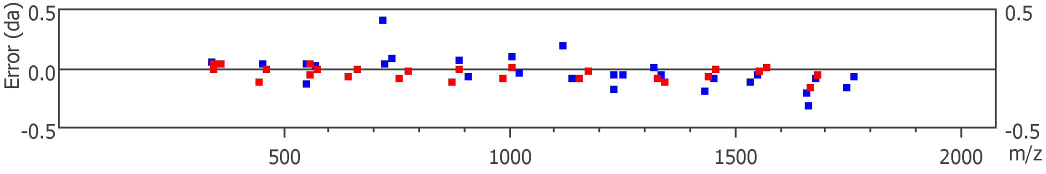


**Mass spectrometry analysis of purified tau expressed in cells identified the doubly acetylated K280/K281 peptide**.

Full-length wild-type Tau-T40 was acetylated by co-expression with CBP followed by immunoprecipitation with anti-tau T14+T46 antibodies, separated by SDS-PAGE, gel excised, and analyzed by mass spectrometry. Listed in the table are the individual ion scores that correspond to the m/z spectrum depicted in Figure 2a. Statistically significant ion scores in red and blue confirm the doubly acetylated K280/K281 peptide, $^{275}$VQIIN<u>**KK**</u>LDLSNVQSK$^{290}$. These data were generated using Mascot software (Matrix Science).

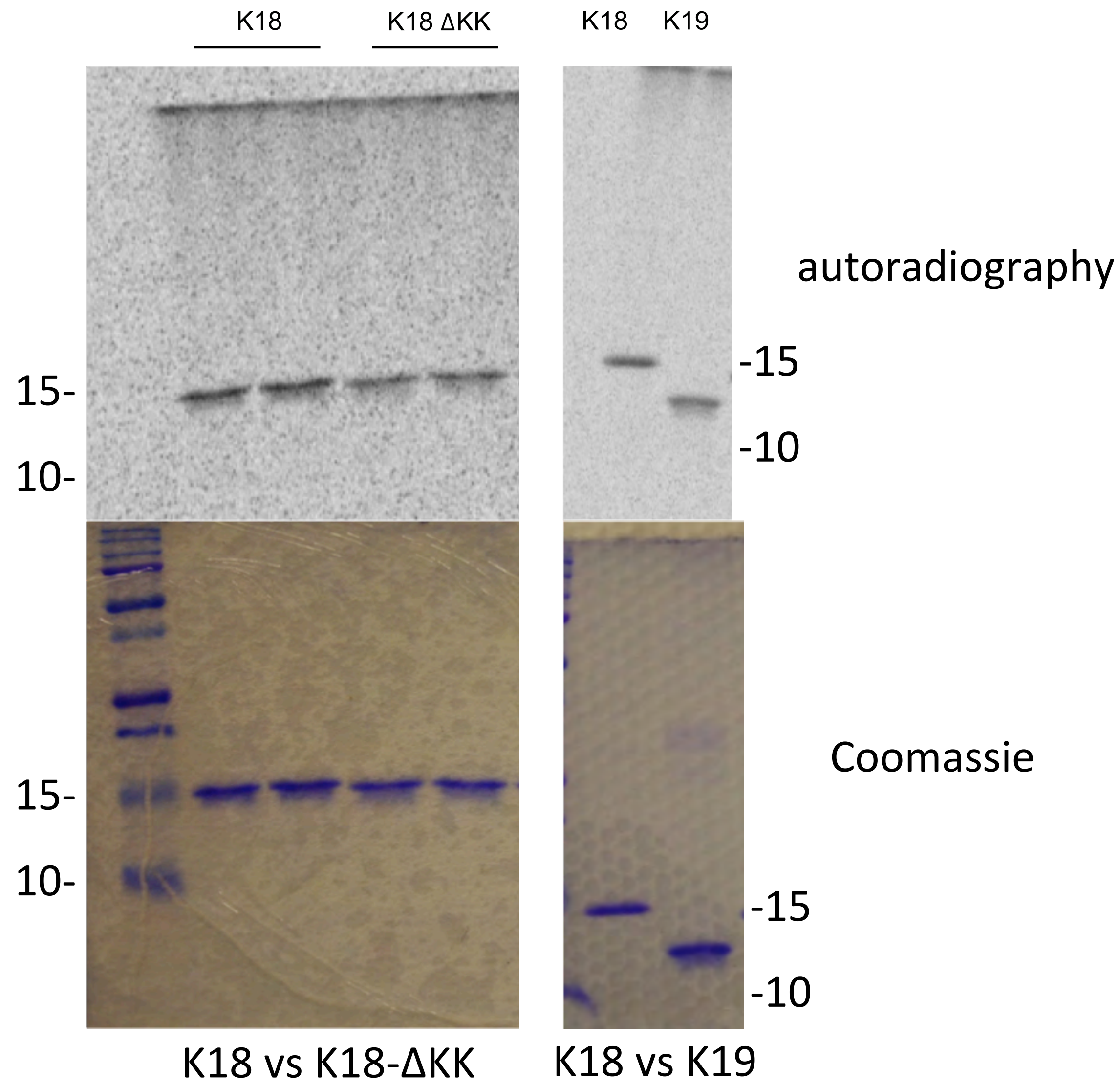


Full-length gels from Fig. 2b and 2c

Trzeciakiewicz et al., Supplementary Figure S4

# Tau K18 fragment

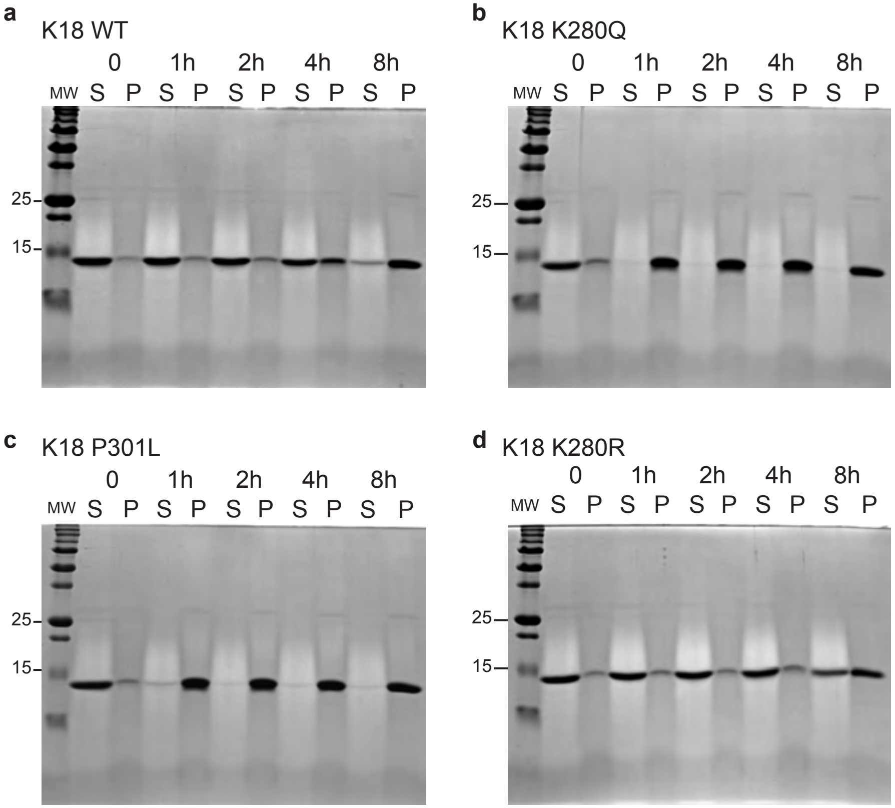


# Full-length tau T40

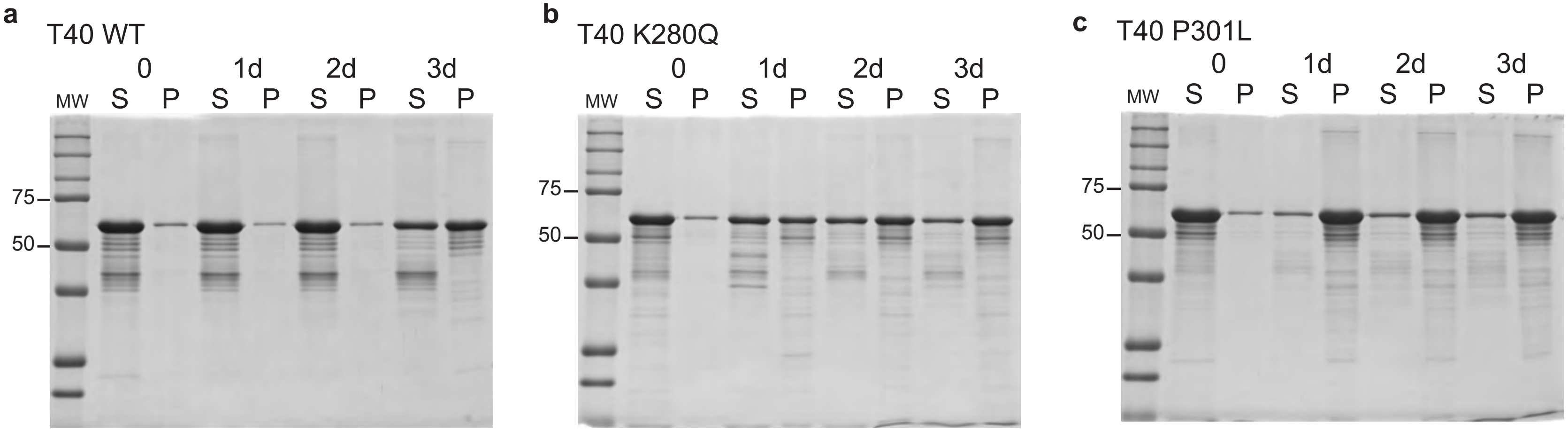


Full-length gels from Fig. 3

Trzeciakiewicz et al., Supplementary Figure S5

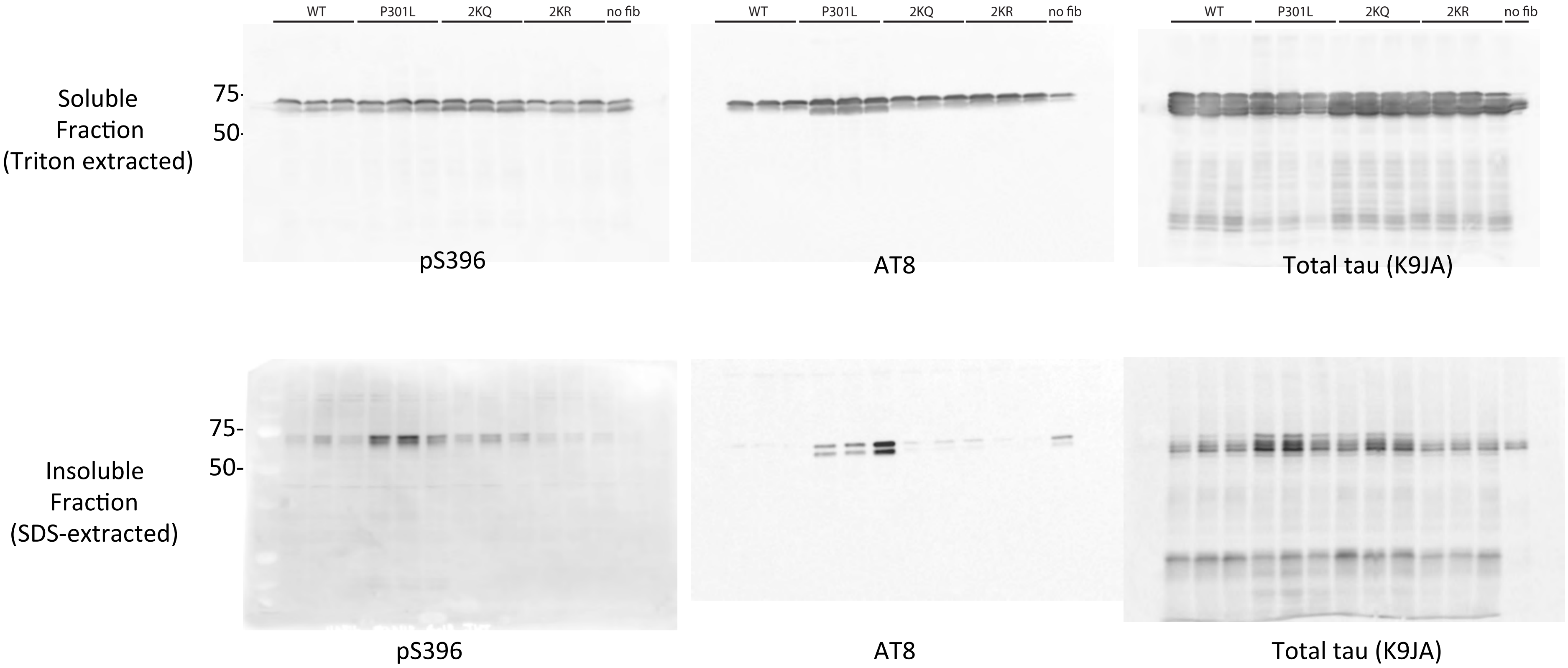


Full-length blots from Fig. 6a

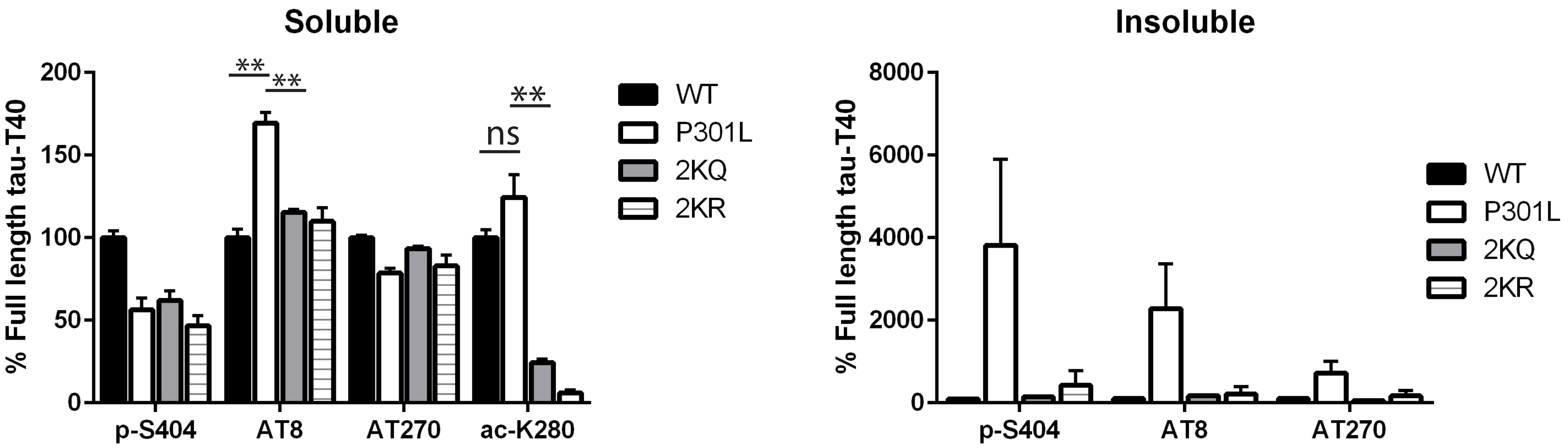


**Tau acetylation mimics enhance seed-dependent tau aggregation in cells with minimal phospho-tau accumulation.** Quantification of immunoblots in Fig. 6a was performed by protein band densitometry. Error bars indicate s.d. of the mean. P-values indicate statistical significance (**, p-value < 0.01).

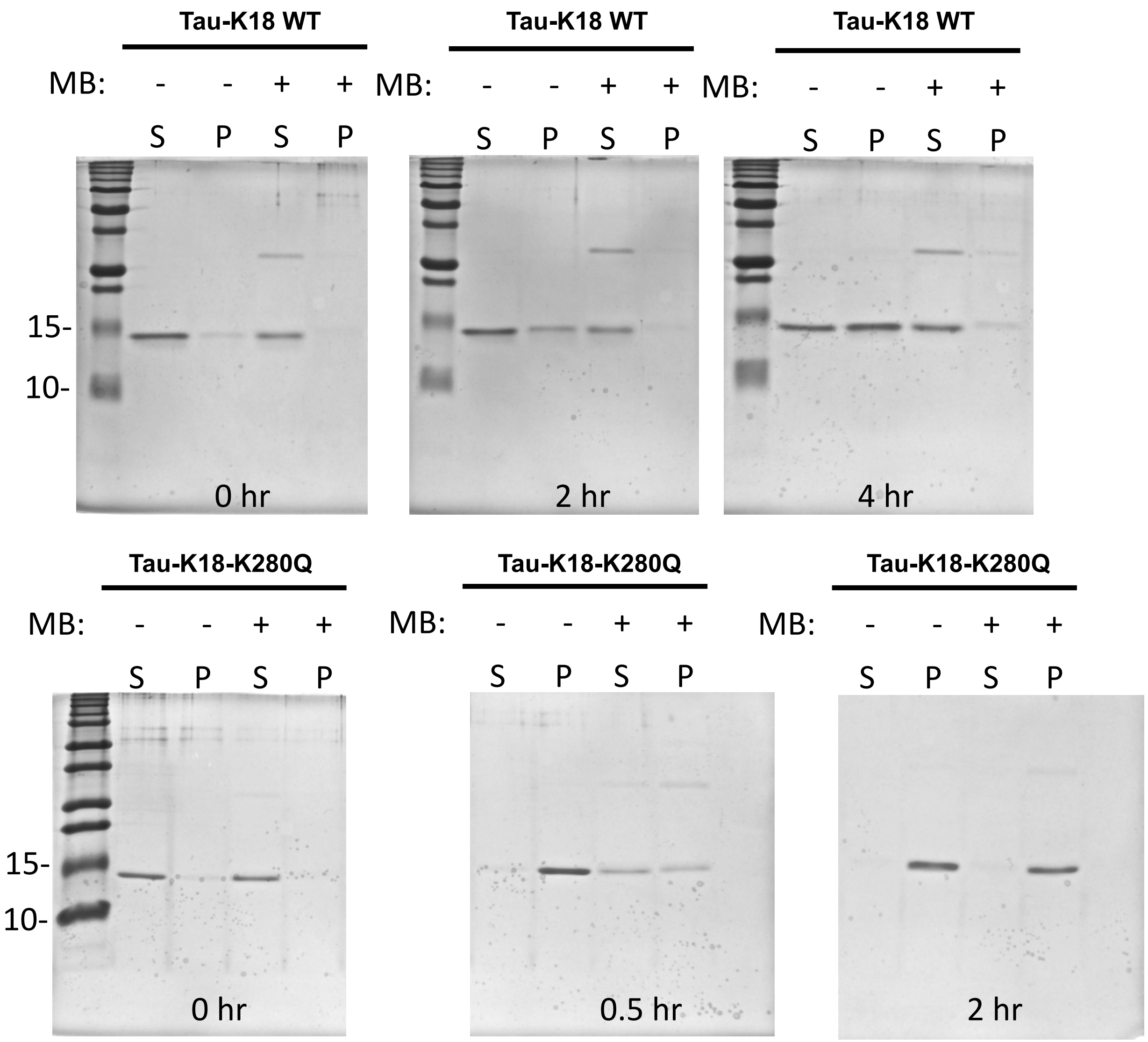


Full-length gels from Fig. 7a

Trzeciakiewicz et al., Supplementary Figure S6

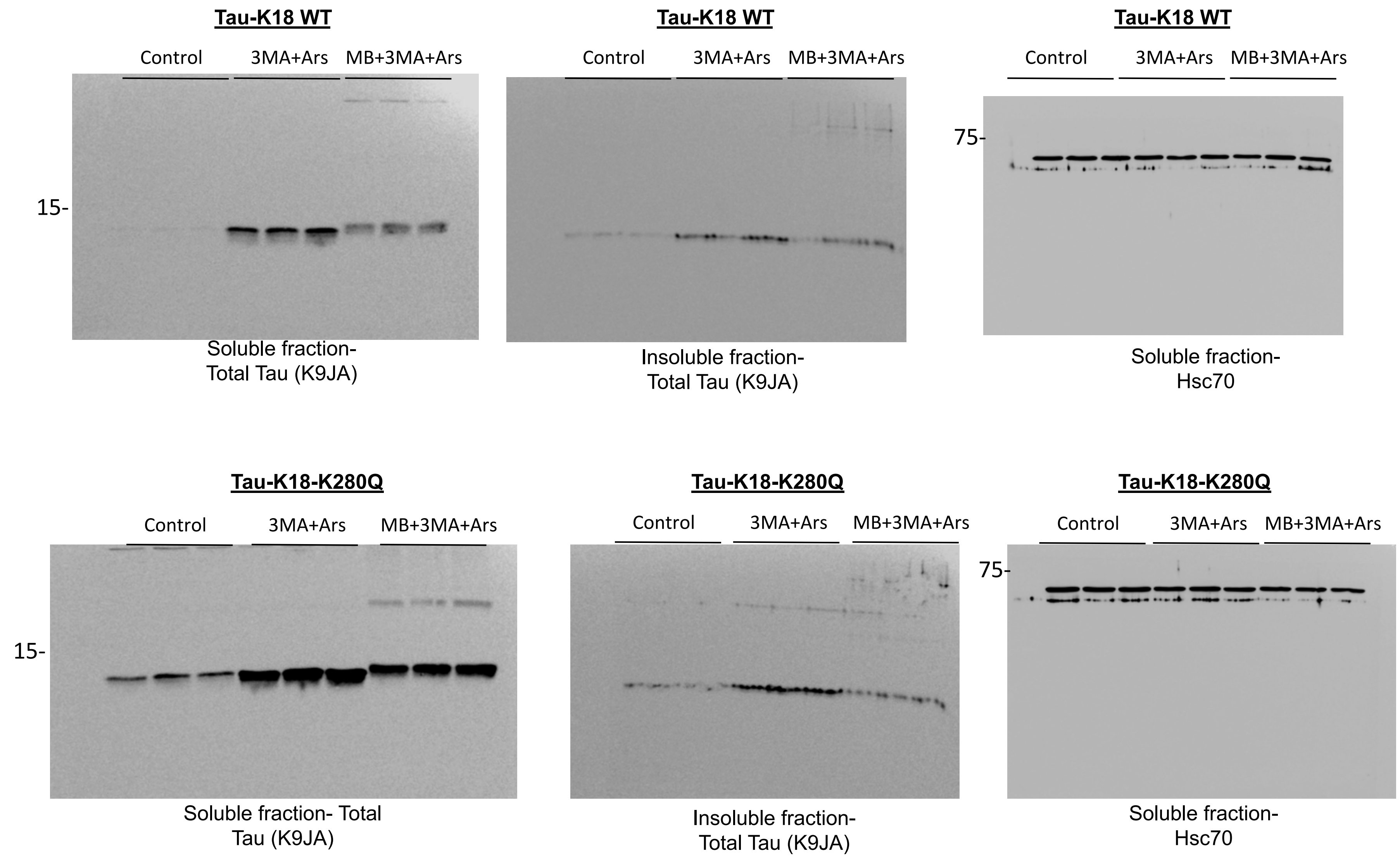


Full-length blots from Fig. 7b

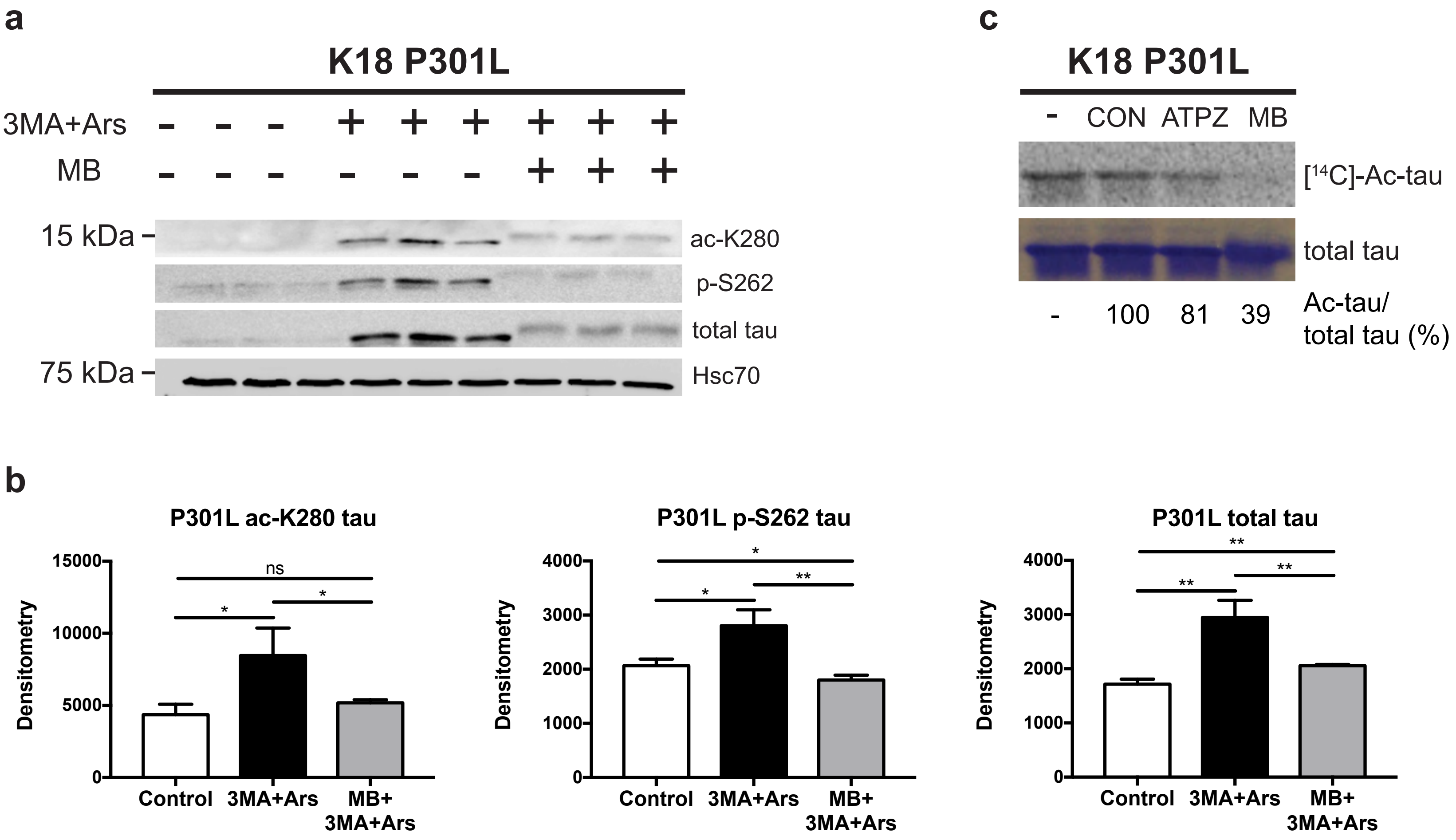


**Methylene blue modulates tau-P301L solubility and acetylation status.**

**a)** Immunoblot analysis with indicated antibodies of cells expressing K18-P301L treated with 3MA, Ars, or MB, where indicated. Hsc70 was included as a loading control. Quantification of immunoblots in **(b)**. Error bars indicate s.d. of the mean (*, $p < 0.05$; **, $p < 0.01$). **c)** Autoradiography and Coomassie blue staining of *in vitro* auto-acetylated recombinant K18-P301L protein incubated with 20 µM inactive control, ATPZ, or MB compounds. The raw intensity ratio of acetylated to total tau protein is presented as a percentage and set relative to untreated control reactions.

Trzeciakiewicz et al., Supplementary Figure S6

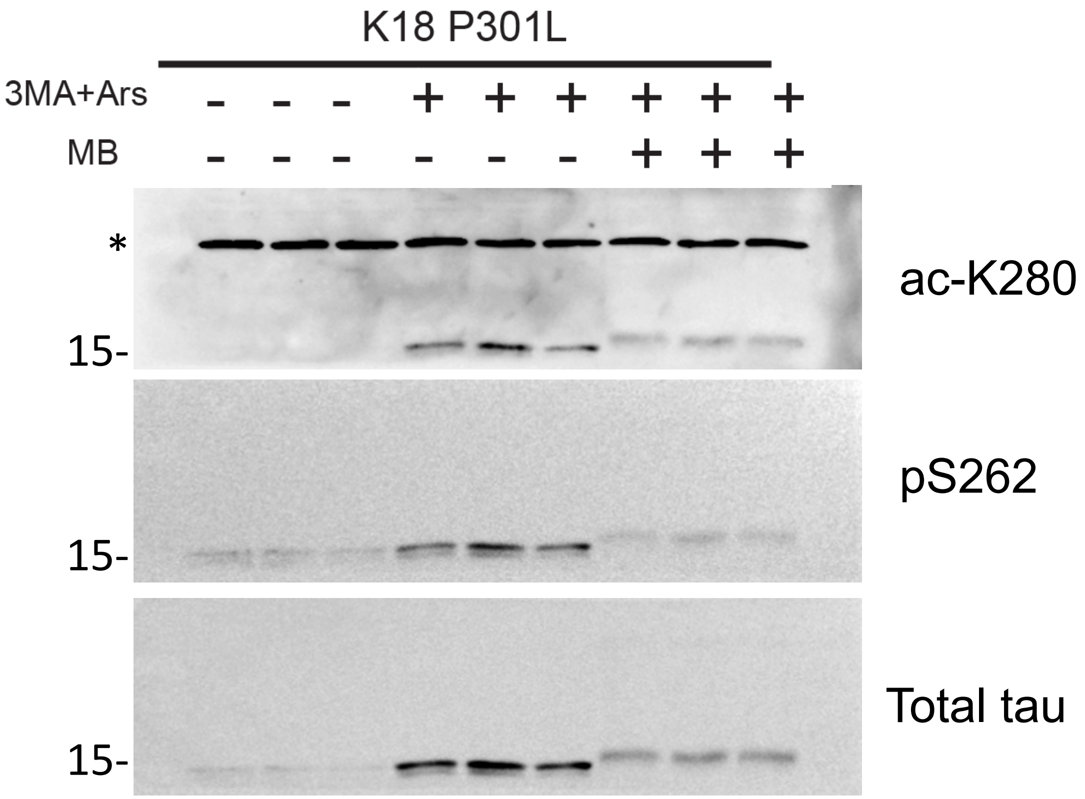


Full-length blots from Supplementary Fig. S6

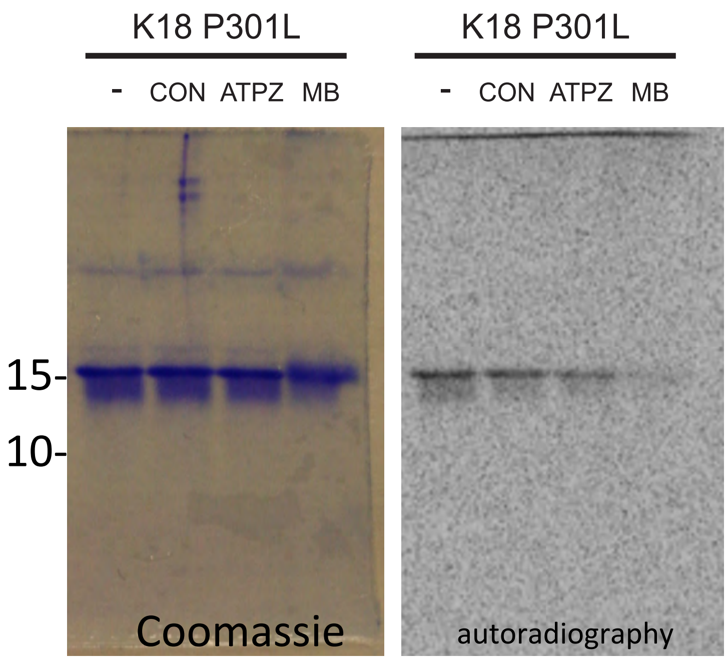


Full-length gels from Supplementary Fig. S6
